# Expression Divergence of Tandemly Arrayed Genes in Human and Mouse

**DOI:** 10.1155/2007/60964

**Published:** 2007-11-06

**Authors:** Valia Shoja, T. M. Murali, Liqing Zhang

**Affiliations:** Department of Computer Science, Virginia Tech, Blacksburg, VA 24060, USA

## Abstract

Tandemly arrayed genes (TAGs) account for about one third of the duplicated genes in eukaryotic genomes, yet there has not been any systematic study of their gene expression patterns. Taking advantage of recently published large-scale microarray data sets, we studied the expression divergence of 361 two-member TAGs in human and 212 two-member TAGs in mouse and examined the effect of sequence divergence, gene orientation, and chromosomal proximity on the divergence of TAG expression patterns. Our results show that there is a weak negative correlation between sequence divergence of TAG members and their expression similarity. There is also a weak negative correlation between chromosomal proximity of TAG members and their expression similarity. We did not detect any significant relationship between gene orientation and expression similarity. We also found that downstream TAG members do not show significantly narrower expression breadth than upstream members, contrary to what we predict based on TAG expression divergence hypothesis that we propose. Finally, we show that both chromosomal proximity and expression correlation in TAGs do not differ significantly from their neighboring non-TAG gene pairs, suggesting that tandem duplication is unlikely to be the cause for the higher-than-random expression association between neighboring genes on a chromosome in human and mouse.

## 1. INTRODUCTION

Gene expression is an important indicator of gene function. Detailed gene function is hard to decipher without many biochemical and physiological experiments. However, the increasing availability of large-scale gene expression profiling makes it much easier to study a gene's function in terms of its expression. Consequently, many important questions on the evolution of gene function have been addressed from the perspective of gene expression. One of the important questions that has benefited from large-scale gene expression data is the evolutionary divergence of gene expression in duplicated genes. To date, two pictures seem to be emerging from these studies. First, divergence of gene expression appears to follow the duplication-degeneration-complementation (DDC) model [1], that is, after duplication, duplicated genes tend to be expressed in different set of tissues, but the total number and types of tissues where duplicated genes are expressed are similar to the counts for the ancestral single-copy gene [2–5]. Second, duplicated genes tend to diverge in expression pattern quickly after duplication [3, 6–8].

However, little is known about evolutionary divergence of gene expression in tandemly arrayed genes (TAGs). These genes are duplicated genes that neighbor each other on a chromosome and account for nearly one third of all duplicated genes in several completed eukaryotic genomes such as human, mouse, rat, worm, Arabidopsis, and rice [9–12]. Thus, studying expression divergence in these genes will provide insights into the functional divergence of a large proportion of duplicated genes.

**Figure 1:**
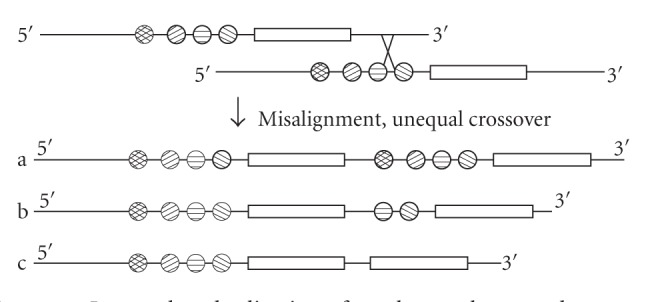
Incomplete duplication of regulatory elements due to unequal crossover. Different regulatory elements are represented by circles with different patterns. Genes are represented by rectangles. Unequal crossover can occur to the left or right side of the gene, in either case, the gene copy that locates upstream will have complete set of regulatory elements, whereas the gene that locates downstream will have complete, partial, or none of the original set of regulatory elements, depending on where the crossover breakpoint occurs. Note that unequal crossover breakpoint can also occur in one of the genes' exonic or intronic regions, which can lead to partial duplication of the gene's exons, but in this study, we only consider complete duplication of all exons of the genes.

To formally study the expression divergence of these tandemly arrayed genes, we suggest a working hypothesis on TAG expression divergence. As TAGs are generated by unequal crossover, depending on the location of crossover points, the downstream member can get either the complete (one extreme, Figure 1(a)), partial (Figure 1(b)), or no regulatory elements (the other extreme, Figure 1(c)) of its ancestral gene. In the last two cases, the downstream member is born with “defects.” To be functional again, it has to capture and obtain upstream regulatory signals for expression. Thus, we expect that the downstream member of a TAG should have a narrower expression breadth than its upstream copy.

In this study, we compiled a list of two-member TAGs in human and mouse. We studied the divergence of TAG gene expression patterns, addressed how expression divergence is affected by sequence divergence, chromosomal proximity, and relative gene orientation, and tested the working hypothesis on TAG expression. Furthermore, it has been shown that neighboring genes have highly correlated gene expression patterns in diverse organisms such as human, worm, fly, yeast, and Arabidoposis [13–16] Tandemly duplicated genes have been thought to be one of the causes for the expression association [14]. Here we contrasted expression association of TAG members with that of their corresponding neighboring gene pairs to examine the interplay of duplication and physical linkage.

## 2. MATERIALS AND METHODS

We retrieved the gene family information and protein sequences for human and mouse from Ensembl (version 39, http://www.ensembl.org). Since information on chromosome location is needed to determine TAGs, only genes with known chromosome locations were kept for further analysis. We used the same method to identify TAGs as in [10]. For the purpose of this study, we considered TAGs with at most one spacer (see Figure 1 in [10]). We limited our study to TAGs of size two because patterns of crossover can be very complex for TAGs with more than two members, which in turn can complicate the interpretation of gene orientations. This restriction did not reduce the number of arrays greatly, as we have shown previously that most TAGs have two members in the array for both human and mouse [10]. Altogether, we obtained 1348 and 1618 TAGs in human and mouse, respectively.

We obtained human and mouse gene expression data from the Gene Expression Atlas (version 2, http://symatlas.gnf.org), which is a collection of gene expression experiments that surveyed the human and mouse transcriptomes in a panel of 79 human and 61 mouse tissues [17]. This study used the Affymetrix HG-U133A array in addition to two custom-made arrays: GNF1H for human and GNF1M for mouse, designed according to human and mouse genome sequences. The results presented here are based on data generated from applying the MAS5 condensation algorithm to the Affymetrix data. The algorithm reports an average difference (AD) value for each gene, which is an estimate of the expression level in a tissue sample [18, 19]. Details of sample annotation and preparation are given in the paper by [17] and at GNF (http://wombat.gnf.org/). Two experimental replicates (samples) for each tissue were obtained in each species. Therefore, we used the average of the two samples for each tissue. To avoid any bias that may be caused by the averaging method, we also randomly picked one of the two AD values for each gene and found that all results were qualitatively the same as using the average values. We therefore report only the results based on the average values.

We used the annotations available in Ensembl and GNF to link TAGs with their probe sets. Probe sets containing probes with higher likelihood of cross-hybridization between genes (Affymetrix IDs indicated by a suffix of “_x_at” or “_s_at”) are considered “suboptimal” reporters of gene expression [3]. For a gene with more than one probe set, if the gene had any higher confidence probe set, we discarded the lower confidence reporters, and took the average of the remaining probe sets. We retained lower confidence reporters if they were the only available probe sets for a gene. We found that most TAGs have either only one gene mapped to a probe set, or none of the two genes linked to probe sets. Discarding these TAGs left us with a total of 361 and 212 TAGs for human and mouse, respectively.

We employed two measurements of tissue specificity. One is *expression breadth*, defined as the number of tissues in which the gene has an AD value of greater than 200, corresponding to ≈3–5 copies per cell [19]. The other is the *tissue specificity index*, τ introduced by [20]. The τ of a specific gene i is

(1) $$\tau_{i}=\frac{\Sigma_{j=1}^{n}\left(1-\log S\left(i,j\right)/\log S_{\max}\left(i\right)\right)}{n-1},$$

where n is the total number of either human or mouse tissues, S(i,j) is the expression of gene i in tissue j, and $S_{\text{max}}\left(i\right)$ is the highest expression signal of gene i across the n tissues. To minimize the influence of noise from low intensities, we let S(i,j) be 100 if it is lower than 100 [21]. The τ value ranges from 0 to 1, with higher values indicating higher tissue specificities. If a gene is equally expressed in all tissues, τ =0. On the other hand, if a gene is only expressed in a few tissues, τ approaches 1.

We used two measures to quantify similarity between the expression profiles of two TAG members: the *Pearson correlation coefficient* (r) and the *Jaccard index* (also known as the Jaccard similarity coefficient). The Jaccard index evaluates the degree of overlap in the types of tissues that two genes are expressed in and is computed using set relations: $J\left(T_{i},T_{j}\right)=|T_{i}\cap T_{j}|/|T_{i}\cup T_{j}|$, where $T_{i}$ and $T_{j}$ are the set of tissues in which genes i and j are expressed, respectively. Thus, the numerator is the number of tissues in which both members of a TAG are expressed while the denominator corresponds to the number of tissues in which at least one member is expressed.

The nucleotide sequences of TAG genes were aligned to each other based on the alignments of corresponding protein sequences using the suite of programs in EMBOSS [22]. The number of synonymous substitutions per synonymous site ($K_{S}$) and the number of nonsynonymous substitutions per nonsynonymous site ($K_{A}$) were calculated using the YN00 program of PAML [23].

There are three kinds of gene orientation in a TAG of size two: parallel orientation when two members are transcribed from the same strand (→→), convergent orientation when two members are transcribed from opposite strands towards each other (→←), and divergent orientation when two members are transcribed from opposite strands away from each other (←→). Sample sizes differ greatly between TAGs with parallel, convergent, and divergent orientations. Therefore, for statistical tests of significance for intergenic distances and comparisons of TAGs and neighboring non-TAGs, we performed the nonparametric Wilcoxon signed-rank tests, as well as bootstrap permutation tests. Specifically, when comparing TAGs in parallel orientations with TAGs in convergent (resp., divergent) orientations, we randomly sampled a subset of parallel TAGs equal in size to the set of TAGs with convergent (resp., divergent) orientations. We calculated the mean of either intergenic distances or expression correlations for this sample. We repeated this random sampling 10 000 times and compared the 10 000 means with that for the other two types of orientation.

## 3. RESULTS

### 3.1. TAG statistics

In human, altogether we identified 361 TAGs of size two, with 247 in parallel, 59 in convergent, and 55 in divergent orientations. In mouse, there are 212 TAGs of size two, with 150 in parallel, 28 in convergent, and 34 in divergent orientations.

### 3.2. Expression divergence

**Figure 2:**
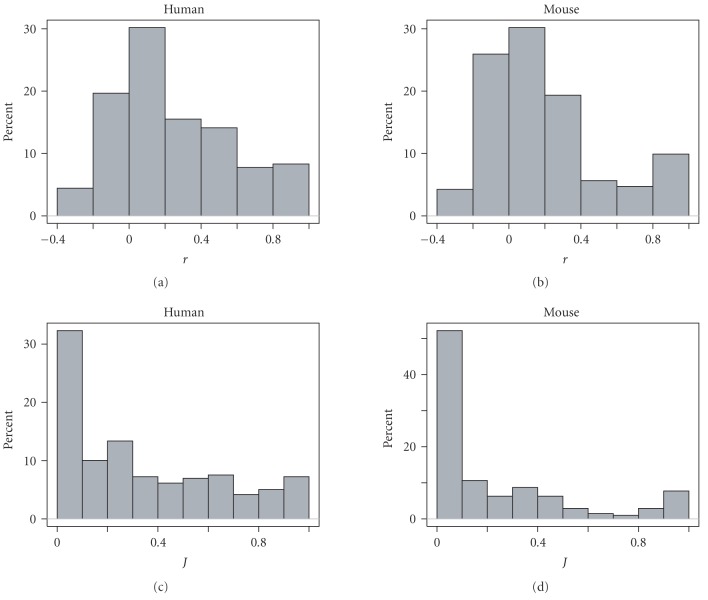
Histogram of two measurements of TAGs' tissue specificity. r is the Pearson correlation coefficient between expression profiles of two TAG members. J is the Jaccard index or Jaccard similarity coefficient of expression profiles of two TAG members. The y-axis denotes the percentage (%) of TAG gene pairs.

Figure 2 shows the distribution of the two measurements of expression similarity between TAG members for all the TAG genes in human and mouse. Both Pearson's r and Jaccard index J show that the majority of human and mouse TAG genes appear to have diverged in expression: 78% of genes in human have r<0.5 and 82% of genes in mouse have r<0.5; 31% of genes in human have J<0.1 and 52% of genes in mouse have J<0.1. Both indices show that mouse seems to have more genes that are diverged in their expression.

**Expression divergence and sequence divergence**

The basic statistics of synonymous ($K_{S}$) and nonsynonymous ($K_{A}$) distances are shown in Table 1. Most of the TAGs are very diverged in their coding sequences as more than 81% of the TAGs in human and 83% of the TAGs in mouse have $K_{S}>1$. The correlation between $K_{S}$ and r is negative but not significant (human: r=-0.06, P-value =.28; mouse: r=-0.04, P-value =.58). The correlation between $K_{A}$ and r is negative but not significant (human: r=-0.04, P-value =.42; mouse: r=-0.0003, P-value =.99).

We also applied restrictions on $K_{S}$ and $K_{A}$ to examine further the correlation between sequence divergence and expression similarity. We used the same criterion as that in [8]. Specifically, limiting gene pairs to those with $K_{S}<1.4$ and $K_{A}<0.7$, we are left with only 75 TAGs. There is a weak negative correlation between expression similarity r and $K_{S}$(r=-0.19, P-value =.096), a weak insignificant correlation between r and $K_{A}$(r=-0.18, P-value =.127), and the correlation becomes much higher when $K_{A}<0.2$(r=-0.30, P-value =.042). Similarly, for mouse, we obtained 35 TAGs. There is a negative yet not significant correlation between r and $K_{S}$(r=-0.15, P-value =.396), and between r and $K_{A}$ when $K_{A}<0.2$(r=-0.42, P-value =.086).

Table 1 also shows sequence divergence for different orientations. Both bootstrap permutation tests and Wilcoxon signed-rank tests show that relative gene orientation in TAGs has little effect on sequence divergence measured by either $K_{S}$ or $K_{A}$ in both species (P-values range from .33 to .88 among all pairwise comparisons of sequence divergence for different orientations).

**Expression divergence and gene orientation**

Table 1 shows the ranges and medians of Pearson's r for gene expression of TAGs in different orientations. For both human and mouse, the medians and ranges of r between TAG members do not differ greatly among different orientations. The bootstrap permutation test as well as the Wilcoxon signed-rank test show that orientation of TAGs has no effect on the expression correlation of TAG members (P-values range from .18 to .91).

**Expression divergence and chromosomal proximity**

Table 1 shows the ranges and medians of intergenic distances between two members of TAGs. When considering all TAGs, we observed a negative correlation between intergenic distances and Pearson's r in human (ρ=-0.15, P-value =.004) but not in mouse (ρ=0.06, P-value =.37). When separating TAGs into groups of different orientations, a negative correlation between intergenic distances and r is observed only for TAGs with parallel orientation in human (ρ=-0.14, P-value =.03).

We also examined the effect of a spacer on expression divergence of TAGs, as spacers effectively increase the intergenic distance between two neighboring TAG members. We defined a spacer as a gene that is in between two TAG members and has a BLASTP E-value greater than $10^{-10}$ to the two TAG members. Since the number of TAGs with one spacer is very small for both human (66) and mouse (37), we performed bootstrap resampling tests and found that TAGs with one spacer show lower expression correlations than TAGs without spacers in human with marginal significance (P-value =.07), but not in mouse (P-value =.9).

**Comparing TAGs to neighboring non-TAGs**

To examine the effect of tandem duplication on expression divergence of neighboring genes, we identified neighboring non-TAG gene pairs either to the immediate left or right side of TAGs, and compared their expression divergences with those of TAGs. We were able to identify 105 neighboring non-TAG pairs in human and 62 in mouse. For these pairs, we calculated the Pearson's correlation coefficients of their gene expression profiles, and then applied paired t-tests to compare expression correlation of the group of TAGs with that of the group of the corresponding neighboring non-TAGs. Results show that expression correlation is not significantly different between the two groups for both species (human: t=1.38, df=104, P-value =.17; mouse: t=1.18, df=61, P-value =.24).

We are also interested in whether TAGs with parallel orientation have shorter intergenic distances than their neighboring non-TAG gene pairs. In both species, the average intergenic distance of neighboring non-TAGs was greater than that of TAGs. However, paired t-tests show that the difference of intergenic distances between TAG and neighboring non-TAG groups is not significant for both species (human: t=-1.66, df=104, P-value =.1; mouse: t=-1.05, df=61, P-value =.3)

**Expression patterns of upstream and downstream genes**

To test the TED hypothesis, we compared the expression patterns of upstream and downstream members of TAGs of parallel orientation both in terms of the number of tissues where they are expressed and tissue specificity. In human, there are 96 TAGs with upstream genes more widely expressed than downstream genes, whereas 139 TAGs have the opposite pattern, and 12 TAGs with equal expression breadth between upstream and downstream members. In mouse, there are 77 TAGs with upstream genes more widely expressed than downstream genes, whereas 65 TAGs with the opposite pattern, and 8 upstream and downstream genes equally widely expressed.

In terms of tissue specificity, in human, there are 103 TAGs with upstream genes less specific than downstream genes, whereas 137 TAGs with the opposite pattern, and 7 upstream and downstream genes with the same tissue specificity. In mouse, there are 76 TAGs with upstream genes less specific than downstream genes, whereas 72 TAGs with the opposite pattern, and 2 upstream and downstream genes with the same tissue specificity.

## 4. Discussion

Gleaning indications on possible divergence of gene functions using expression data have become a routine practice in understanding the evolution of duplicated genes (e.g., [3, 6–8, 24]). For instance, [7] examined 400 duplicate gene pairs in yeast for their expression divergence using microarray data and found that more than 40% of the gene pairs in the study show diverged expression pattern even when $K_{S}<0.1$ and more than 80% for $K_{S}<1.5$. Similarly, [8] showed that of the 1404 duplicate gene pairs that they studied in human, more than 73% show diverged expression in at least one tissue when $K_{S}<0.064$; the number increases to 90% for $K_{S}<1.2$. Therefore, both studies suggest that expression patterns of duplicate genes diverge rapidly after duplication. Furthermore, both studies show that expression similarity is significantly negatively correlated with $K_{S}$.

In addition, [7] found that there is a weak correlation between the Pearson correlation coefficient of the expression profiles and $K_{A}$ when $K_{A}<0.7$. This negative correlation becomes much higher for $K_{A}<0.3$. They noted that the 0.3 selection is arbitrary and used two other values ($K_{A}<0.25$ and $K_{A}<0.35$) and found a similar negative correlation. Similarly, [8] also found a weak but significant negative correlation between expression similarity and $K_{A}$ for $K_{A}<0.7$ in the human data, the negative correlation becoming stronger when limiting the dataset to gene pairs with $K_{A}<0.2$. Taken together, the two studies in yeast and human suggest that expression divergence and protein sequence divergence are coupled shortly after gene duplication.

Contrary to the findings of Li's group, in an earlier study, [24] found no significant correlation between expression divergence and protein sequence divergence in 144 yeast duplicated genes. The data in Wagner's study was the expression of 144 duplicated genes measured at multiple time points in 4 physiological processes in yeast, compared to the microarray data from 14 processes for 400 gene pairs in [7]. Thus, it seems most likely that the data in Wagner's study was too small to detect any statistical significance.

In fact, Wagner's study seems to provide a good analogy to our study since we also did not find any significant correlation between expression divergence and sequence divergence in TAGs, unlike the study of [8]. One difference between the studies is that we used the microarray data produced by [17] in 2004, while Makova and Li used an earlier data produced by the same research group [19]. However, this is unlikely to be the main reason for the discrepancy between the two studies.

Further analyses of TAGs with different $K_{S}$ and $K_{A}$ thresholds suggest that our result is largely consistent with what previous studies have found regarding the correlation between expression similarity and sequence divergence in duplicated genes (see results). However, most of the correlations in our study are not statistically significant, which is most likely due to the small sample sizes (75 TAG gene pairs in our study versus 1404 in the study of Makova and Li). Moreover, we noted that the negative correlation coefficients (albeit not significant) shown by either the 75 TAGs in human or 35 TAGs in mouse are much higher than those computed on the entire dataset, suggesting that expression divergence of duplicated genes (including TAGs) and their sequence divergence are strongly coupled only shortly after duplication.

[25, 26] pointed out that the standard model of gene duplication assumes an exact duplication of the ancestral gene, whereas in reality, partial duplication along with exon shuffling and gene fusion may also be common and affect the ultimate fate of the newly arising duplicate. They compared the exon-intron structure of duplicated genes and found that more than 50% of the duplicated copies exhibit gene structural divergence when $K_{S}=0$ and this number increases to about 60% when $K_{S}<0.1$. The actual proportion of incomplete duplications could be even higher as only exon-intron structures were compared between duplicated genes in their studies. Their observation shows that it is common that the new gene is born without all the exons that its ancestral copy has.

Considering the complexity of gene structure, it is not difficult to imagine that incomplete duplication can also happen at the regulatory regions of a gene, in which case, only some portions of the promoter elements of the ancestral copy are duplicated and inherited by the newly arising copy. In TAGs, partial duplication can be achieved mechanistically through unequal crossover as illustrated in Figure 1. If a crossover occurs somewhere in the middle of promoter regions, the downstream gene may get only part of or none of the regulatory elements that the upstream copy has and is thus born “crippled” in terms of how widely it is expressed. In the extreme case where the downstream gene is born without any regulatory elements, it has to capture promoter elements from somewhere upstream of its coding region. The gene's initial expression capacity thus depends heavily on how many regulatory elements it inherits. Taking this phenomenon into consideration, our working hypothesis on TAG expression posits that since incomplete duplication in regulatory regions can result in fewer regulatory motifs in downstream genes than their upstream genes, and because null mutations occur equally likely in the regulatory elements of both upstream and downstream copies, downstream genes are expected to have, on average, a narrower expression breadth and higher tissue specificity than their upstream copies.

**Figure 3:**
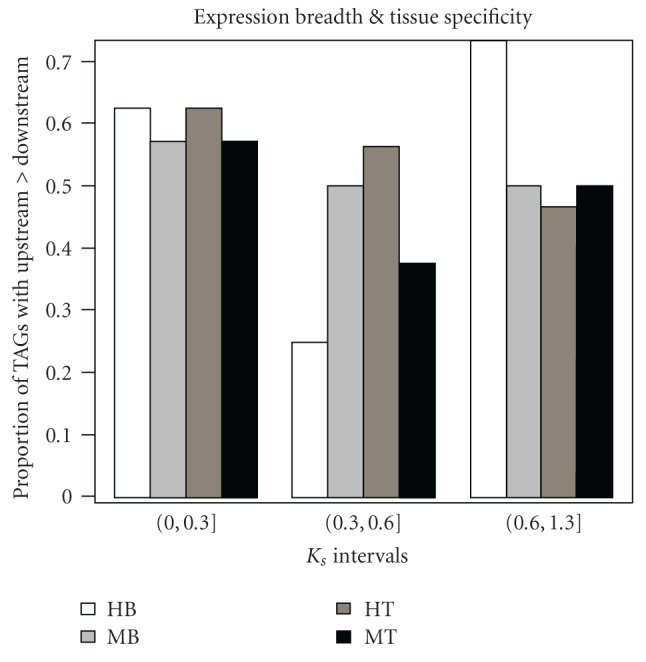
Proportion of TAGs with upstream genes more widely expressed than downstream copies in three $K_{S}$ divergence groups, based on two measurements (expression breadth and tissue specificity). HB and MB are results using breadth measurements in human and mouse, respectively; HT and MT are results using tissue specificity in human and mouse, respectively.

There are two explanations for why our observations do not support the working hypothesis. First, an important factor that can influence our results substantially is the age of the TAGs. Even if downstream genes did not inherit any regulatory elements at the onset of duplication, given sufficient time, they might obtain upstream regulatory motifs and become expressed in different tissues during evolution. In fact, capturing upstream signal for expression has been reported in a number of cases such as retrotransposed genes [27]. In order to examine whether age has an effect on our prediction, we grouped TAGs into low, medium, and high divergence groups based on $K_{S}$ and calculated for each group the proportion of TAGs that have upstream genes more widely expressed than downstream ones. We considered only TAGs with divergence of $K_{S}<1.3$ in both human and mouse. There are altogether 47 TAGs in human and 27 TAGs in mouse that satisfy this criterion. The low, medium, and high divergences correspond to $K_{S}$ intervals of (0,0.3], (0.3,0.6], and (0.6,1.3], respectively. These bins were chosen to obtain roughly the same number of genes in each $K_{S}$ interval. Altogether, the low, medium, and high divergences groups contain 16, 16, and 15 TAGs in human, respectively, and 7, 8, and 12 TAGs in mouse, respectively. Based on the breadth measurement (i.e., the number of tissues in which genes are expressed), the proportions of TAGs that have upstream genes more widely expressed than downstream genes for the three divergence groups are 62.5%, 25.0%, 73.3% in human, respectively, and 57.1%, 50.0%, 50.0% in mouse, respectively. Based on the tissue specificity index, the proportions become 62.5% 56.3%, 46.7% in human, respectively, and 57.1%, 37.5%, and 50.0% in mouse, respectively (Figure 3). Therefore, it seems that in recently duplicated TAGs, there is a higher proportion of TAGs that bear our prediction, and as evolution progresses, some of the downstream gene might have obtained novel regulatory elements and gained new expression patterns, which in turn led to the decrease in the proportion of TAGs in which upstream genes are more widely expressed than downstream ones. As the distribution of the Jaccard Index shows in Figure 2, most of the TAG members share little overlap in the tissues where they are expressed, suggesting the possibility that some downstream genes might have indeed obtained new regulatory motifs after duplication.

Second, an important assumption implied in the working hypothesis is that duplication does not necessarily contain the complete set of regulatory elements. However, if most or all of tandem duplications include the entirety of upstream motifs, we expect no particular patterns as to which copy should be more widely expressed because the downstream copy is equally likely to be more widely expressed than the upstream one or vice versa. The observation that the intergenic distances between TAG members range from 47 bp (base pairs) to 4.3 Mbp (mega bp) in human, and from 160 bp to 0.9 Mbp in mouse with a median of 23 Kbp (kilo bp) and 21 Kbp in the two species, respectively (Table 1), suggests that many tandem duplications that generated these TAGs might have included the complete set of regulatory elements. We note that noise in microarray data is unlikely to be a major reason, since noise should affect both upstream genes and downstream genes equally.

Gene expression is highly correlated between neighboring genes on a chromosome in organisms such as human [28], *C. elegans* [14, 29], yeast [13], fly [15], and *A. thaliana* [16]. However, different mechanisms seem to be responsible for the correlation. For example, in the *C. elegans* genome, tandem duplication seems to be especially common and removing tandem duplicates reduces the degree of expression correlation in neighboring genes [14, 29]. In yeast, the coexpression of neighboring genes seems to be determined by higher-order structures such as chromosomal domain level controlled expression activity [13]. In this paper, the comparison of the expression correlation of TAG gene pairs with that of their neighboring non-TAG gene pairs shows that neighboring genes that arose from tandem duplication do not have significantly higher expression correlation than ones that did not arise from tandem duplication (see Section 3), suggesting that tandem duplication is unlikely to be the cause for the higher-than-random expression association between neighboring genes in human.

Studies of expression correlation between neighboring genes also include the exploration of factors such as intergenic distance and gene orientation that maybe influence the expression correlation of neighboring genes (e.g., [13, 14, 16, 29]). Both factors seem to play a role in affecting the degree of expression association between neighboring genes. TAGs are special cases of neighboring genes as they share sequence similarity due to duplication. Consistent with previous studies, our results show that intergenic distance between TAG members seems to play a role in determining the expression divergence of TAGs, at least in human. However, the orientation of TAG gene pairs seems to have no effect on their expression correlation. Interestingly, [13] have shown that although divergent gene pairs show highest expression correlation among the three types of orientation, the difference in expression correlation disappears when gene pairs in different orientations are required to have similar intergenic distances. Consistent with this finding, our results show that neither the intergenic distances nor the expression correlations of TAGs with different orientations are statistically different from each other. Taken together, the results seem to suggest that global (chromatin) effects on expression regulation of the TAGs are more important than local factors such as gene orientation and local regulatory elements.

## Figures and Tables

**Table 1:** TAG sequence divergence ($K_{S}$ and $K_{A}$), expression correlation, and intergenic distances (Kb) in different orientations.

		Human			Mouse		
	Orientation	Lower	Median	Upper	Lower	Median	Upper
		quartile		quartile	quartile		quartile
Sequence	Parallel	1.41	5.69	64.40	1.58	8.25	63.84
divergence	Convergent	1.18	3.81	65.94	2.02	41.67	67.32
$K_{S}$	Divergent	1.85	32.35	71.33	2.23	11.81	60.47
	All	1.37	7.35	64.81	1.54	8.31	64.47
Sequence	Parallel	0.29	0.44	0.61	0.28	0.46	0.63
divergence	Convergent	0.19	0.39	0.54	0.25	0.45	0.59
$K_{A}$	Divergent	0.26	0.48	0.63	0.44	0.51	0.66
	All	0.27	0.44	0.61	0.29	0.48	0.63
Expression	Parallel	0.02	0.17	0.45	−0.03	0.11	0.36
correlation	Convergent	0.05	0.19	0.60	−0.03	0.09	0.35
	Divergent	0.06	0.19	0.58	0.05	0.10	0.24
	All	0.01	0.18	0.46	−0.02	0.11	0.35
Intergenic	Parallel	7.99	18.61	39.88	6.48	15.00	32.21
distance	Convergent	8.61	19.02	31.45	5.85	20.40	42.66
	Divergent	7.12	23.09	79.32	17.00	27.29	48.99
	All	9.35	23.21	51.60	8.70	21.14	47.35
